# Dental Science

**Published:** 1850-01

**Authors:** 


### DENTAL SCIENCE.

DR. TAYLOR,—DEAR SIR.—During a recess in our late meeting in Louisville, the question was agitated:

Has dental science attained to the highest degree of excellence of which it is susceptible? Has it been carried as near to perfection as its votaries should hope and strive to place it? At that time I thought it more prudent in me to hear than to speak; but the question has frequently recurred to my mind since, and I feel disposed to say something on the subject at present, more especially because my views differ materially from any that I have ever heard publicly expressed on this point. While some insist that the science of dentistry, (so far as filling teeth is concerned) has not made any advancement, since Hudson blistered his hand in using foil of unusual thickness.

And that so far as the insertion of teeth is concerned, the plan of inserting Porcelain teeth on gold plate, admits of no improvement, at least so far as the character of the materials employed are concerned, and that all that is left for the dentist of the present day to do, is to follow in the footsteps of his illustrious predecessors. Others flatter themselves that the indomitable energy and boundless enterprise, guided by the revelations of chemistry,of aspirants for distingnished and superior excellence in dental operations will ultimately advance the science so far beyond its present circumscribed limits, that the decay or loss of the teeth will, in reality, be no loss at all, except in a pecuniary sense. In other words that the art of filling decayed teeth will attain such a degree of perfection, that both the appearance, utility and durability may be secured in their pristine integrity; and that artificial substitutes will be inserted on an improved plan that will secure very nearly, if not all the advantages to those who may have occasion to employ them, that accrue to those who are so fortunate as to retain their natural teeth.

Now the result of my own speculations and calculations differs widely from both these extremes, (as I regard them.) Though Dr. Hudson doubtless filled some teeth in the best possible manner, and with the best possible material, that does not prove that the art or science admits of no further improvement. If the pioneer setlers in our western country succeeded in raising large crops of sound corn on the rich virgin soil of the Miami bottoms,does that prove that the science of agriculture admitted of no further improvement, even in the cultivation of this important product?

On the contrary, abundant crops may now be raised on lands that at that time were thought to be not worth cultivation; and the quality of the soil may be annually improved under a judicious course of treatment, instead of rapidly exhausting it as the former method of cultivation was calculated to do. And

I doubt not likewise that many diseased teeth may now be treated with decided success, that Dr. Hudson and his contemporaries would not have attempted to treat at all, and that all the common operations that occur in the practice of dentistry can be performed as well in every respect, (and in much less time) by hundreds of dentists throughout the country, as they were performed by those eminent and estimable men who first labored to advance and elevate their favorite art to the advantagious position that it now occupies, and in regard to the insertion of artificial teeth. Though the use of porcelain teeth, mounted on gold plate should never be superceeded by materials better adapted to this purpose; still the method and style of constructing and applying them has been so greatly improved already, that I doubt whether the individual who first employed these materials, if he could see a specimen of the most perfect workmanship in this department now in use, would have the assurance to lay claim to the invention. But whether human ingenuity and research will ever be able to invent or discover any materials better adapted to supply the wants of the dentist—to preserve or supply the place of the natural teeth, or not. I have no doubt but important improvements may still be made in the modes of operation, in the construction of instruments, and also in the skill, dexterity and precision with which they shall be employed.

But before all those advantages can be fully realized, the human constitution must be more perfectly developed and equally balanced, and this will be calculated to do away the necessity for many of the operations that would otherwise be required; and render permanent many that would otherwise be frail and require frequent renewal.

So that the science of dentistry can never be advanced to the highest degree of excellence and perfection of which it is susceptible, otherwise than by the employment of means, that must of necessity, prove fatal to its existence.

And to me, the enterprise appears far more practicable and probable, that means for the effectual preservation of the natural teeth will be discovered, and thus put a most effectual stop to the further progress of the dental art, rather than that it can ever be brought to such a degree of perfection as to enable those who practice it, to compete successfully with the handi-work of nature; besides being a much more desirable consummation, even if both were equally easy of access. I hope I shall not be regarded as recreant to the highest interest of a profession of which I claim to be an humble member, or of a science of which I profess to be a votary, when I declare that I regard it as one of my most important and indispensible duties as a dentist; to inquire dilligently, to ascertain as far as practicable, and point out as clearly as possible what are the best means of preserving the teeth from decay; and arresting the various diseases to which they are liable in their incipient stages.

In other words, I regard the relation that I bear to my race, as taking precedence of that which I bear to the fraternity of dental surgeons; the former was bestowed on me by the Creator without any choice or volition of my own, the latter was voluntarily assumed, the former must continue while man exists on earth, the latter will of necessity shortly terminate.

And farther I claim not only connection with, but an interest in posterity; and if I can aid in conferring on the future representatives of that interest, a sound constitution, or even a perfect and permanent set of teeth, I should contribute more towards the promotion of their real advantage, in such a boon than in the bestowment of pecuniary treasures beyond computation. However others may regard such a sentiment or principle, I declare most candidly and unequivocally that my chief inducement to pursue the labors of a dentist, is not the acquisition of wealth, (though I trust that I estimate the advantages of wealth at their sure value,) but because I believe that I may contribute more to the general welfare of our race in this, than in any other employment by which I could secure a competence for the support of myself and those dependent on me; and if it was in my power to do so, most gladly would I avert the necessity for any further dental operations, by giving to every individual of our race a sound and perfect set of teeth, with the disposition and skill necessary to preserve them in such condition. But this may not be done instantaneously, but I believe may be effected gradually, not by directing our attention exclusively to the teeth; but by bestowing the necessary care on the whole constitution together. And I regard the opportunities enjoyed by dentists for forwarding this great and glorious enterprise as being equal, if not superior to those enjoyed by any class of men in existence.

M., of Augusta, Ky.

